# Empirical Support for Optimal Virulence in a Castrating Parasite

**DOI:** 10.1371/journal.pbio.0040197

**Published:** 2006-05-30

**Authors:** Knut Helge Jensen, Tom Little, Arne Skorping, Dieter Ebert

**Affiliations:** **1**Department of Biology, University of Fribourg, Fribourg, Switzerland; **2**Department of Biology, University of Bergen, Bergen, Norway; **3**The School of Biological Sciences, University of Edinburgh, Edinburgh, Scotland, United Kingdom; **4**Zoologisches Institut, University of Basel, Basel, Switzerland; Pennsylvania State UniversityUnited States of America

## Abstract

The trade-off hypothesis for the evolution of virulence predicts that parasite transmission stage production and host exploitation are balanced such that lifetime transmission success (LTS) is maximised. However, the experimental evidence for this prediction is weak, mainly because LTS, which indicates parasite fitness, has been difficult to measure. For castrating parasites, this simple model has been modified to take into account that parasites convert host reproductive resources into transmission stages. Parasites that kill the host too early will hardly benefit from these resources, while postponing the killing of the host results in diminished returns. As predicted from optimality models, a parasite inducing castration should therefore castrate early, but show intermediate levels of virulence, where virulence is measured as time to host killing. We studied virulence in an experimental system where a bacterial parasite castrates its host and produces spores that are not released until after host death. This permits estimating the LTS of the parasite, which can then be related to its virulence. We exposed replicate individual Daphnia magna (Crustacea) of one host clone to the same amount of bacterial spores and followed individuals until their death. We found that the parasite shows strong variation in the time to kill its host and that transmission stage production peaks at an intermediate level of virulence. A further experiment tested for the genetic basis of variation in virulence by comparing survival curves of daphniids infected with parasite spores obtained from early killing versus late killing infections. Hosts infected with early killer spores had a significantly higher death rate as compared to those infected with late killers, indicating that variation in time to death was at least in part caused by genetic differences among parasites. We speculate that the clear peak in lifetime reproductive success at intermediate killing times may be caused by the exceptionally strong physiological trade-off between host and parasite reproduction. This is the first experimental study to demonstrate that the production of propagules is highest at intermediate levels of virulence and that parasite genetic variability is available to drive the evolution of virulence in this system.

## Abstract 2

Exposing replicate *Daphnia* hosts to the same amount of bacterial spores from the castrating bacterium *Pasteuria ramose* provides experimental evidence that parasite fitness is maximized at intermediate levels of virulence.

## Introduction

Current theory on the evolution of virulence is based on the assumption that there is a trade-off between different parasite fitness components [ 1– 4]. For example, a higher parasite reproductive rate will usually increase transmission, but this can only be achieved by harming the host more, thereby decreasing host (and parasite) longevity. Thus, increased virulence can sometimes increase parasite fitness, but parasite fitness will likely be maximised at a point that is below the maximum virulence level. This led Anderson and May [ 1] to propose that under such trade-off conditions, parasite fitness is optimised at intermediate levels of virulence.

This model has been used to explain a wide range of phenomena related to virulence, such as coevolution between hosts and parasites [ 5], evolutionary effects of drugs and vaccines [ 6], and the spread of emergent diseases [ 7]. Although there is empirical support for a trade-off between host exploitation and host survival [ 8, 9], and a positive genetic relationship between virulence and transmission (e.g., [ 10]), direct evidence that the transmission success is maximal at intermediate levels of virulence is lacking. To test for the existence of an optimal level of virulence, it is necessary to determine the relationship between virulence and the cumulative production of transmission stages over the entire lifetime of an infection.

For castrating parasites, the trade-off model has been modified to incorporate the parasite's capacity to convert host reproductive resources into transmission stages [ 11– 13]. These models predict that the optimal level of castration is total castration, but make no specific predictions for time to host death. Ebert et al. [ 14] produced a verbal model to take into account that many castrators also induce enhanced growth of their hosts (gigantism) (e.g. [ 15– 19]). It was suggested that gigantism benefits the parasite, as it allows the storage of castration-liberated host resources into host body mass until the parasite can make use of them. The parasite may benefit from the larger host size as a bigger host provides more resources to the parasite. Castrating parasites usually have high resource demands, as they can reach considerable biomass (often 25% of host biomass) [ 15], which creates a strong negative correlation between parasite and host reproduction [ 14]. Premature host death would not allow efficient exploitation of the host, while postponing the killing of the host would result in diminished returns, as the growth trajectory of the host levels off with time. Analogous to the classical optimality models for virulence [ 1– 4], a parasite inducing castration and gigantism should therefore show intermediate levels of virulence, where virulence is measured as time to host killing. The strong physiological trade-off between parasite reproduction and time to host death makes castrating parasites strong candidates to visualise the relationship between parasite fitness and virulence.

The castrating bacterium Pasteuria ramosa produces spores that accumulate in large numbers within the host and are not released until after the host has died, and thus the number of spores at host death is a good estimate of parasite lifetime reproduction [ 20]. To test for a virulence optimum, we exposed individual daphniids of a single host clone to bacterial spores and followed each individual until its death. To verify if variation in time to host death has a genetic basis in parasites, we conducted a further experiment in which we infected hosts with parasite spores obtained from early killing and late killing infections.

## Results/Discussion

Infected *Daphnia* showed substantially lower survivorship than either unexposed controls or daphniids that were exposed to the parasite but remained uninfected ( Figure 1). Among infected hosts, the first and last death occurred at ages 23 and 74 d, respectively (median = 51, interquartile range = 31), while the first control animal died at an age of 96 d ( Figure 1). Ten of 39 infected hosts died before the parasite was able to produce any spores, while none of the 71 unexposed control hosts died during the same period. Thus, early killing is clearly detrimental for the parasite, because the development of the transmission spores of P. ramosa takes several weeks. Host castration took place earlier, where infected hosts produced between one and three clutches and ceased reproducing at ages between 11 and 19 d. Total reproduction before castration varied between 5 and 23 offspring (mean = 12.3), and is strongly positively correlated with age at castration (Spearman correlation, rho = 0.764, *p* < 0.001). Thus, age at castration determines fitness for infected hosts.

**Figure 1:**
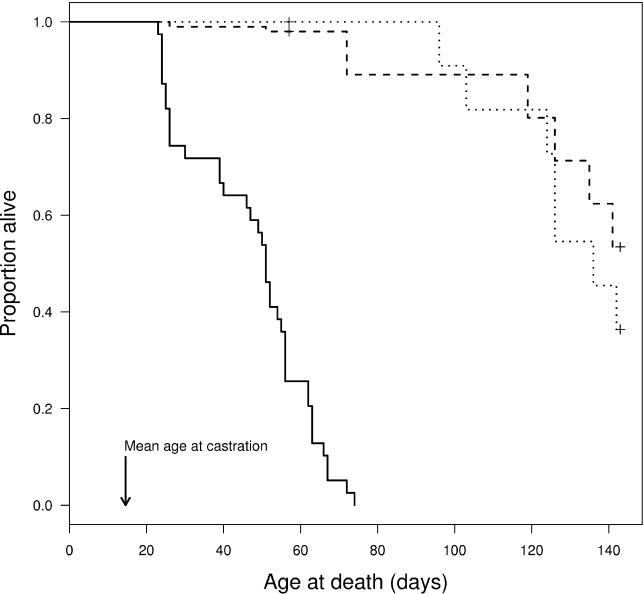
Proportion of Hosts Surviving over Time Depending on Treatment Solid, dashed, and dotted lines represent infected, exposed but uninfected, and unexposed control daphniids, respectively. Survival analysis showed a highly significant difference in survival between the infected as compared to unexposed control hosts ( *p* < 0.001), while there was no difference between the two groups of uninfected daphniids ( *p* = 0.719). The + symbols at the two curves representing uninfected daphniids indicate points where censoring was performed. For more details about the statistics, see Materials and Methods. Mean age at castration is the mean age at which infected animals became castrated. Age at castration is defined as the first day after a host's last reproduction. All infected daphniids had become castrated at an age of 19 d (17 d after infection + 2 d).

For each infected host we counted the total number of mature spores produced and compared this with the time to host death. Daphniids surviving to between 55 and 60 d contained the highest numbers of bacterial spores ( Figure 2). Hosts dying earlier or later than this had a lower number of spores. Thus, both bacteria with a low level of virulence (killing the host late) as well as those causing high virulence (killing the host early) had a lower life time spore production as compared to those killing at an intermediate time span. As total spore production should be closely correlated with parasite fitness our data indicate that an intermediate level of virulence gives the highest fitness for this parasite.

**Figure 2:**
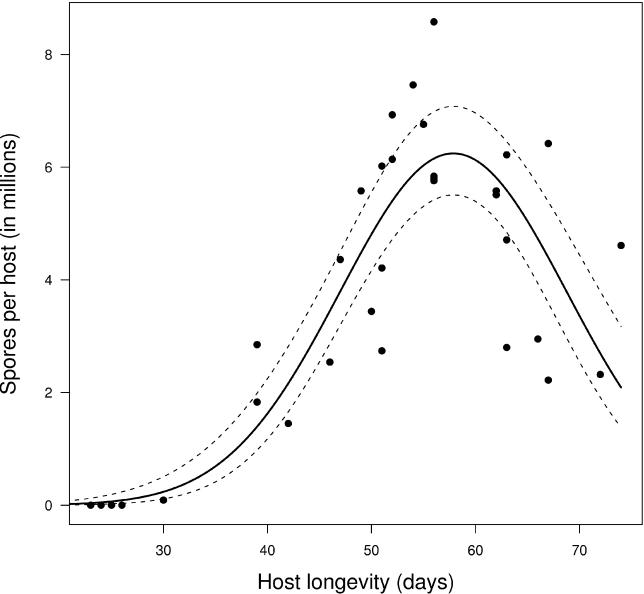
Longevity of D. magna Depending on Spore Production of P. ramosa The total number of infected females is 39 ( *n* = 39 data points in the figure). The solid curve represents predicted values from a generalised linear model with best fit (lowest residual deviance). The dashed curves represent 95% confidence interval for the fitted model curve. For more details about the statistics, see Materials and Methods.

Unintentional, stochastic variation in spore dose may contribute to the observed results to some degree, but since all daphniids in the experiments received the same dose under the same conditions, this variation is likely to be small. Certainly much smaller than the several orders of magnitude in spore dose variation, which would be necessary to create the observed variability in spore load and host death [ 21]. In the framework of the evolution of virulence it is necessary to show that genetic differences among parasite genotypes correlate with differences in parasite fitness and virulence. To test if genetic variation among parasite lines within our P. ramosa isolate contributed to the observed variation in virulence, we conducted a further experiment. To test this we infected daphniids with spores obtained from hosts which were killed early (but in a different experiment than the one shown above) and compared them to hosts infected with spores obtained from hosts killed late. Consistent with our hypothesis, spores from early killing infections caused a significantly higher death rate than late killer spores ( Figure 3). This shows that genetic variation in P. ramosa can underlie variability in time to host killing, and thus the potential to drive the evolution of virulence.

**Figure 3:**
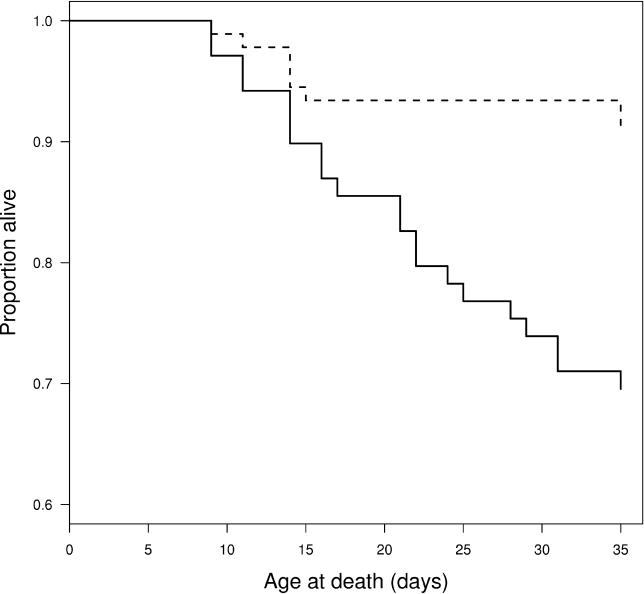
The Genetic Basis of Virulence in P. ramosa The solid and dashed lines show survival of daphniids infected with secondary spores from early and late killing P. ramosa, respectively. The secondary spores were produced in a previous experiment and were extracted from hosts killed by the parasite at 25 to 37 d and 55 to 67 d of age, respectively. There is a significant difference in survival between daphniids infected with early or late killing spores ( *p* < 0.001). For more details about the statistics, see Materials and Methods.

P. ramosa must go through a series of developmental stages before the fully developed transmission spores are produced [ 22], explaining the low spore yields associated with early host death. The following increase in spore yield with increasing age at death could be related to the benefits of using host resources for parasite spore production. It has been estimated that each clutch of host eggs is equivalent to 4.5 million P. ramosa spores [ 14]. A more prudent rate of parasite growth and possibly slower rate of development could allow parasites to harvest more of the resources liberated through host castration. On the other hand, parasites killing the host beyond a certain age showed a decline in total number of spores. Excessively slow growth of the parasite may not lead to efficient host exploitation, and old hosts may be a poor resource for the parasite.

The trade-off between parasite fitness components in our study system is similar to the assumed trade-off between transmission rate and parasite induced host mortality in non-castrator systems [ 1– 4] in that prudent host exploitation strategies result in a reduction of parasite lifetime transmission success. Our castrating parasite differs from other systems in that early killing results in reduced transmission success because of an apparent constraint in spore development. The classical virulence model [ 1– 4] does not include (but does not explicitly exclude) a minimal time required for parasite development. However, our finding of a humped-shaped transmission success-virulence relationship holds even if we exclude those infections, which resulted in premature parasite death. It would be interesting to determine if the minimum time required for spore development can be reduced under conditions which favour earlier host death.

In the present study, the time for the parasite to kill its host spanned 51 d. Another study of the same system tested under similar conditions, but with naturally sympatric antagonists, showed a variability in host killing of approximately 20 d, although the mean age at death was similar [ 14]. Our choice of a novel host clone-parasite isolate combination may have resulted in a relatively high variability in virulence and therefore increased the possibility of detecting an optimum level of virulence. In particular, premature host killing is rare in co-evolved *Daphnia-Pasteuria* combinations [ 14, 21, 23]. Expression of hidden genetic variation in life history traits is known to be associated with changed conditions [ 24– 26].

Infected daphniids were larger than uninfecteds throughout their life, but the growth curves for each group levelled off around the same time, which is around the optimum time of killing ( Figure 4). In a model about the optimal virulence for obligately killing noncastrating parasites [ 27], killing was suggested to be optimal around the time the growth trajectory levels off shortly after host maturity. In the current system, this idea may be extended to include the liberation of resources caused by castration. Parasite-induced gigantism may be a strategy to make use of resources which are liberated at times the parasites has comparatively little energy demands, i.e., at times it has a small biomass [ 14].

**Figure 4:**
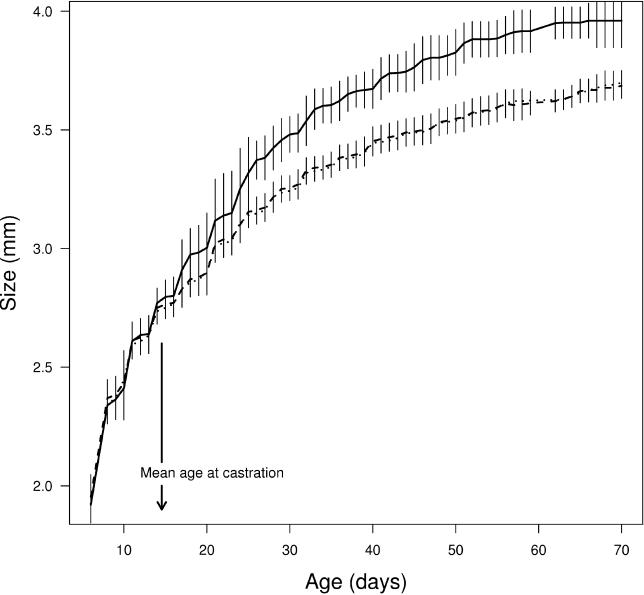
Growth of Genetically Identical D. magna Females Depending on Infection with the Castrating Bacterium P. ramosa Solid, dashed, and dotted lines represent mean size of infected, exposed but not infected, and unexposed control daphniids at the given ages, respectively. The error bars represent ±1 standard deviation of mean size at the given ages. The number of replicates in each treatment is infected ( *n* = 39), exposed but not infected ( *n* = 102), and unexposed control ( *n* = 71). Nonlinear generalised least-squares models for the growth curves showed that the asymptotic size of infected daphniids were significantly larger than both the unexposed controls and exposed but not infecteds, while the asymptotic size of two latter groups did not differ. This shows that only infections can induce host gigantism while parasite exposure alone is insufficient in doing so. For more details about the statistics, see Materials and Methods. Mean age at castration is explained in Figure 1.

Our results strongly indicate that there is an optimum level of virulence for P. ramosa infecting D. magna. This clear visibility of the fitness maximum may be due to the strong physiological trade-off between host and parasite fitness, mediated through competition for resources needed for host and parasite reproduction. This finding supports the idea that trade-offs can play a role in the evolution of virulence. We plan to carry out further experiments to explore the tempo and mode of virulence change under selection. This will enable estimation of the long-term, evolutionary changes in parasite virulence caused, for example, by changes in host demography or by drugs or other controlling agents that alter parasite fitness components and demography [ 4, 6].

## Materials and Methods

The two D. magna clones used were isolated from a pond near Gaarzerfeld, Northern Germany (clone DG-1–106) and from a pond in Regent's Park, London, United Kingdom (clone EL-75–69). The P. ramosa used was extracted from a single infected D. magna collected near Gaarzerfeld, northern Germany. The host female produced viable offspring before the parasite curtailed host reproduction. These offspring were used to produce clone DG-1–106. The original infected female was kept in the laboratory until she died, at which point her cadaver was crushed and then used to infect new hosts derived from her genetically identical offspring. Thus, the parasite spores isolated from a single wild-caught female were propagated only on her own isofemale line. To build up parasite numbers for experimentation, this propagation was repeated as described in Little et al. [ 28]. Briefly, each new round of infections was performed in 200-ml jars with ten hosts per jar and there were ten replicate jars per passage. Infections were always initiated with 1 × 10 ^6^ spores per jar and infection periods were 7 d. Infected hosts were grown until they died, at which point they were pooled and frozen for use in the next passage. The parasite was not cloned or bottlenecked during this procedure.

Two hundred twelve D. magna females (clone EL-75–69) born within 18 h were placed singly in glass vials and kept in a climate chamber under standardised laboratory conditions with artificial culture medium [ 29], a temperature of 19 °C, and a 16:8 h light/dark cycle. At an age of 2 d, 141 of the juveniles were each exposed to 2 × 10 ^4^ spores of P. ramosa that were extracted from many host individuals (DG-1–106), while the rest (71) were exposed to placebo made from the same amount of ground up *Daphnia* tissue (same clone) as used when making spore solution, but from uninfected daphniids. The latter group was a control to compare growth, reproduction, and survival with the parasite exposed daphniids. The level of water in each glass vial was 20 ml until 4 d after exposure, when water was changed and increased to 70 ml in each vial and changed every third day thereafter. Before the first water change, the daphniids were fed with 3 million cells of the green algae Scenedesmus sp. each day and thereafter with 5 million cells each day. After 4 d of parasite exposure, size of the daphniids was measured under a stereomicroscope on a daily basis, except at ages 8, 60, and 61 d, respectively. Since daphniids mainly grow during moulting, each individual was measured only after moulting. This was detected from a free-floating carapace in the vial, which happens approximately every third day, except for early in life when moulting takes place more frequently. The number of parasite spores in infected hosts was counted at the day of death (only for the 15 earliest killed daphniids) or the host was frozen at the day of death and spores counted at a later time. Spores were counted by first grinding up the host and then using a single-celled counting chamber with Thoma ruling. To reduce the workload with the controls towards the end of the experiment, we performed a random removal of replicates at day 57 within the two groups of uninfected daphniids. The number of replicates was reduced to 11 within each of the two uninfected groups, while ten of the infected hosts still were alive.

The genetic basis for variation in virulence was tested in an independent experiment. The source of parasite spores was the same as above (clone DG-1–106). An experiment was conducted using spores from infected hosts that had either died early (day 25 to 37; termed early killers) or late (day 55 to 67; termed late killers). Early or late killers were used to infect fresh hosts (clone DG-1–106) to determine if their rate of killing is a stably inherited trait. For this, we performed infections as above, except there were 12 independent replicates for each spore type and spore dose was 1 × 10 ^5^ spores per jar. The proportion of hosts surviving in each jar was monitored until the daphniids reached an age of 35 d.

All statistics were performed by using the R statistical package, version 2.1.1 [ 30]. To test for a difference between the survival curves in Figure 1, we performed survival analyses by using the survreg function. The syntax for the model giving the lowest residual deviance was s *urvreg(Surv(age.at.death, status) ˜ category, dist = 'extreme')*, where *category* is a variable with the three levels; infected, exposed but not infected, and unexposed controls, respectively, and *status* is a censoring variable where dead animals are coded as 1 and animals removed from the experiment at day 57 (see above) or animals still alive at the end of the experiment were coded as 0. The curve in Figure 2 was created from a generalised linear model calculated by using the glm function with the following model syntax: *glm(Spores ˜ poly(Longevity,2), quasipoisson)*. This model explains significantly more of the deviance in the data as compared to the same model without the second order polynomial ( *F* = 111.2, *df* = 1, *p* < 0.001). The experiment testing the genetic basis for variation in virulence ( Figure 3) was analysed by using a mixed model repeated-measurements ANOVA to account for a random effect of jars. We did this on the arcsin square-root transformed proportion of hosts surviving in each jar per day. The syntax for the model was: lme(proportion.alive ˜ category * age, random = ˜+1|jar, cor = corAR1()), where *category* is the variable with the two levels; early and late killing spores, respectively. The growth curves ( Figure 4) were tested against each other by using the gnls function for nonlinear generalised least squares models. Syntax for the models were: gnls(size ˜ Sinf*(1 − *exp(*− K*(age − *t0))), params = Sinf + K + t0 ˜ 1, start = list(Sinf = 5,K = 0.1,t0 = 0), control = list(returnObject = T), corr = corCAR1(form = ˜age | replicate, fixed = T))*, where *size* = size at age *i*, *Sinf* = asymptotic size, K = curvature parameter, age = age *i*, and t0 = age of size 0. The resulting asymptotic sizes of infected, exposed but not infected, and unexposed control daphniids (where numbers in parentheses represent lower and upper 95% confidence limits) were 3.96 (3.93, 4.00), 3.63 (3.62, 3.64), and 3.63 (3.62, 3.64), respectively. Curves with nonoverlapping confidence intervals of the asymptotic sizes were determined to be significantly different.
